# Shorter telomeres in children with severe asthma, an indicative of accelerated aging

**DOI:** 10.18632/aging.202527

**Published:** 2021-01-20

**Authors:** Florencia M. Barbé-Tuana, Lucas K. Grun, Vinícius Pierdoná, Mariana M. Parisi, Frederico Friedrich, Fátima T.C.R. Guma, Leonardo A. Pinto, Renato T. Stein, Paulo M.C. Pitrez, Marcus H. Jones

**Affiliations:** 1 Group of Inflammation and Cellular Senescence, Laboratory of Immunobiology, School of Health, Sciences and Life, Pontifical Catholic University of Rio Grande do Sul (PUCRS), Porto Alegre, Brazil; 2 Postgraduate Program in Cellular and Molecular Biology, School of Health, Sciences and Life, Pontifical Catholic University of Rio Grande do Sul (PUCRS), Porto Alegre, Brazil; 3 Postgraduate Program in Pediatrics and Child Health, School of Medicine, Pontifical Catholic University of Rio Grande do Sul (PUCRS), Porto Alegre, Brazil; 4 Laboratory of Respiratory Physiology, Infant Center, School of Medicine, Pontifical Catholic University of Rio Grande do Sul (PUCRS), Porto Alegre, Brazil; 5 Postgraduate Program in Biological Sciences, Biochemistry, Federal University of Rio Grande do Sul (UFRGS), Porto Alegre, Brazil; 6 Group of Comprehensive Health Care, Centre for Health and Rural Sciences, University of Cruz Alta, Cruz Alta, Brazil; 7 Pediatric Pulmonology Division, Hospital Moinhos de Vento, Porto Alegre, Brazil

**Keywords:** telomere length, CCL11, severe asthma, inflammaging, senescence

## Abstract

Severe therapy-resistant asthma (STRA) is closely associated with distinct clinical and inflammatory pheno-endotypes, which may contribute to the development of age-related comorbidities. Evidence has demonstrated a contribution of accelerated telomere shortening on the poor prognosis of respiratory diseases in adults. Eotaxin-1 (CCL11) is an important chemokine for eosinophilic recruitment and the progression of asthma. In the last years has also been proposed as an age-promoting factor. This study aimed to investigate the association of relative telomere length (rTL) and eotaxin-1 in asthmatic children. Children aged 8-14 years (n=267) were classified as healthy control (HC, n=126), mild asthma (MA, n=124) or severe therapy-resistant asthma (STRA, n=17). rTL was performed by qPCR from peripheral blood. Eotaxin-1 was quantified by ELISA from fresh-frozen plasma. STRA had shorter telomeres compared to HC (*p*=0.02) and MA (*p*=0.006). Eotaxin-1 levels were up-regulated in STRA [median; IQR25-75)] [(1,190 pg/mL; 108–2,510)] compared to MA [(638 pg/mL; 134–1,460)] (*p*=0.03) or HC [(627 pg/mL; 108–1,750)] (*p*<0.01). Additionally, shorter telomeres were inversely correlated with eotaxin-1 levels in STRA (r=-0.6, *p*=0.013). Our results suggest that short telomeres and up-regulated eotaxin-1, features of accelerated aging, could prematurely contribute to a senescent phenotype increasing the risk for early development of age-related diseases in asthma.

## INTRODUCTION

Asthma is a complex, heterogeneous, and prevalent chronic respiratory disease that can develop early during childhood and persist through life. It is characterized by variable airflow obstruction and tissue remodeling of the airways. Allergic asthma, one of its common phenotypes, often begins in childhood and is characterized by the accumulation of eosinophils in the airways with overproduction of reactive oxygen species (ROS). Eotaxin-1, also called CC motif chemokine ligand 11 (CCL11), is a potent selective chemoattractant for eosinophils. Independent of important functions in the persistent phenotype of allergy and asthma, eotaxin-1 is linked to functions throughout the body. In the central nervous system, the eotaxin-1 expression is associated with cognitive dysfunction, reduced neurogenesis, impaired memory, and learning, all features observed in aging. Administration of eotaxin-1 in young mice recapitulates cognitive impairment observed in old animals. Because eotaxin-1 is elevated in aged mice and humans it has been identified as an aging-associated factor [1]. Accelerated shortening of the telomeres, the repetitive tandem sequences localized at the termini of linear eukaryotic chromosomes, anticipates the aging process leading to diseases. Adults with asthma present short telomeres and there is an association between accelerated telomeres shortening and disease severity [2]. Individuals with persistent or severe asthma, where continuous activation of the immune cells and eosinophilic activity is not satisfactorily controlled have shorter telomeres than healthy individuals [3]. Taken together, these studies suggest that telomere length (TL) analysis could work as a predictor of disease progression in respiratory diseases. This may be crucial if observed in severe diseases from very early in life. As a hypothetical model, the evaluation of TL in children with severe therapy-resistant asthma (STRA) as a predictor of senescence might be clinically useful. In this work, we investigated differences in TL and eotaxin-1 in children with STRA and compared it to mild asthmatics (MA), and healthy controls (HC).

## RESULTS

**Figure 1:**
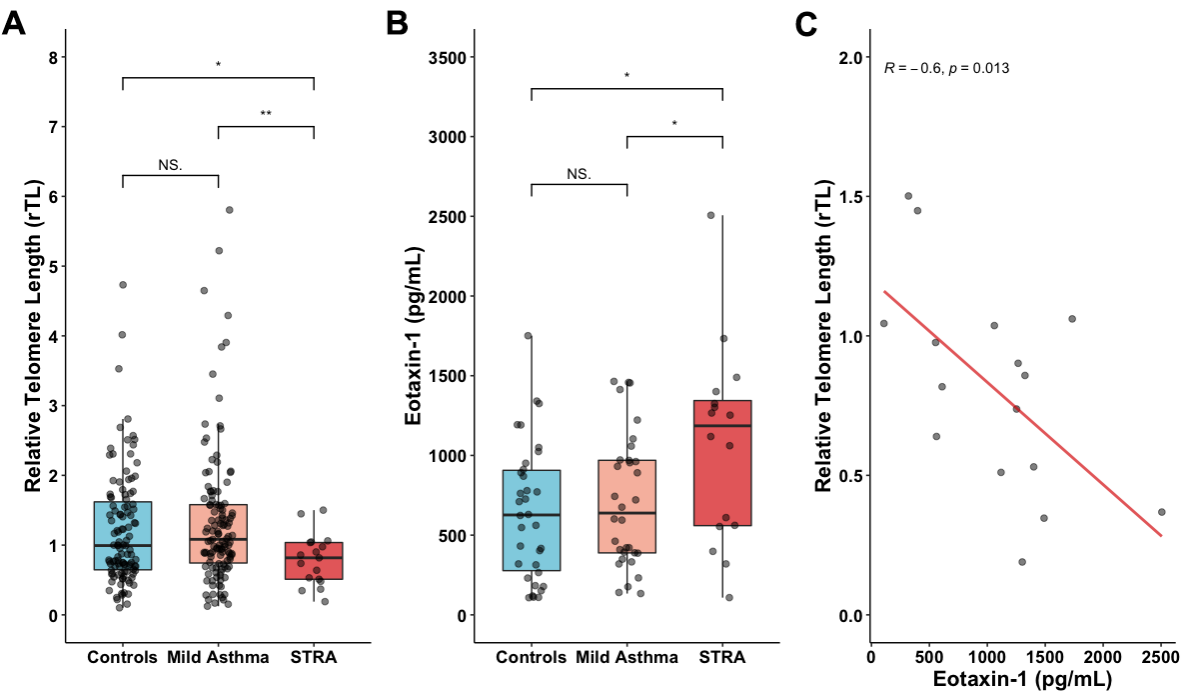
**Shorter telomeres in STRA children.** (**A**) Relative telomere length (rTL) measured in whole blood from HC (n=126), MA (n=124) and STRA (n=17). (**B**) Augmented levels of eotaxin-1 in STRA children (n=16) compared to HC (n=24) and MA (n=24). (**C**) Eotaxin-1 is inversely correlated with rTL only in the STRA group. Data presented as [median; IQR (25-75)]. Kruskal-Wallis test followed by Dunn's multiple comparisons and Pearson correlation test. Significant differences were considered when *p*<0.05 (*); *p*<0.01 (**). NS: non-significant.

Demographic data of HC, MA and STRA patients are presented in Table 1. The STRA group had lower height than HC and MA, but no difference was observed in age (*p*=0.080), weight (p=0.108), proportion of ethnicity (p=0.176) or sex (*p*=0.359) between groups. Children with STRA had significantly shorter rTL [median; IQR(25-75)] [0.818; (0.189–1.50)] when compared to HC [0.993; (0.101–4.73] (*p*=0.02) and MA [1.08; (0.124–5.80)] (*p*=0.006) (Figure 1A), after adjusting for age, sex, or height. A detailed description of the STRA group is shown in Table 2.

**Table 1:** Demographic and clinical characteristics of the subjects*.

**Characteristics**	**Controls, HC** **(n=126)**	**Mild asthma, MA** **(n=124)**	**Severe asthma, STRA (n=17)**	**p**
Median age (IQR) years	11.1(10.0, 11.9)	11.1(10.6, 11.7)	9.5(6.8, 12.0)^ab^	0.080
Median height (IQR) cm	146(139, 152)	147(140, 151)	129(126, 145)^ab^	**0.006**
Median weight (IQR) Kg	39.7(34.0, 48.8)	40.8(35.2, 48.6)	39(26.5, 42.8)^b^	0.108
Female sex n (%)	74(59%)	62(50%)	10(59%)	0.359
Race n (%)				0.176
White	100(79%)	79 (64%)	11 (65%)	
Black	12(10%)	23 (19%)	2 (12%)	
Mixed	14(11%)	22 (18%)	4 (24%)	
Spirometry (Z score)				
Median FVC (IQR)	0.23 (-0.35, 0.73)	0.18 (-0.17, 0.77)	0.62 (0.05, 1.19)	0.487
Median FEV1 (IQR)	0.04 (-0.53, 0.59)	-0.08 (-0.79, 0.48)	0.71 (-0.87, 0.98)	0.302
Median FEV1/FVC (IQR)	-0.54 (-1.09, 0.02)	-0.89 (-1.77, -0.17)c	-1.19 (-2.08, -0.02)^a^	**0.013**
Median FEF25-75 (IQR)	-0.11 (-0.77, 0.45)	-0.54 (-1.36, 0.24)c	-0.54 (-2.28, 0.24)^a^	**0.011**
Median FEF25-75/FVC (IQR)	-0.30 (-0.62, 0.23)	-0.62 (-1.24, -0.01)c	-0.97 (-2.12, -0.40)^a^	**0.001**

*Percentages may not total 100 because of rounding. Race was reported by patients. *p*-values were calculated by means of Kruskal-Wallis test for quantitative variables and Pearson’s Chi-square test for qualitative variables.

^a^*p*<0.05 for the comparison between STRA and Controls by Dunn's Multiple Comparison Test.

^b^*p*<0.05 for the comparison between STRA and Mild Asthma by Dunn's Multiple Comparison Test.

^c^*p*<0.05 for the comparison between Mild Asthma and Controls by Dunn's Multiple Comparison Test.

**Table 2:** Clinical features and laboratory characteristics of the STRA group.

Family History of Asthma n (%)	12/16; (85.7%)
Allergic Rhinitis n (%)	17/17; (100.0%)
Atopic Dermatitis n (%)	4/16; (25.0%)
Smoke Exposure at Home (yes/no) n (%)	7/14; (50.0%)
Body Mass Index Classification^a^	
Healthy Weight	8/16; (50.0%)
Overweight	3/16; (18.8%)
Obese	5/16; (31.2%)
Age of asthma onset (years)	14/17; 0.95 [0.50, 1.75]
Length of follow-up (years)	15/17; 2.00 [1.25, 3.00]
Asthma hospitalizations in the last 12 months	
None	6/15; (40.0%)
1 or 2 hospitalizations	3/15; (20.0%)
3 or more hospitalizations	6/15; (40.0%)
Treatment with Omalizumab	7/16; (43.8%)
IgE total (IU/mL)	16/17; 708.50 [364, 2,000]
Eosinophils (cells/uL)	11/17; 384 [131, 725]
Telomere length (unit)	17/17; 0.82 [0.51, 1.04]
Eotaxin-1 (pg/mL)	16/17; 2,371 [1,119; 2,688]

IU: International Units; STRA: Severe therapy resistant asthma.

^a^Classification: Healthy weight is defined as -2 to 1 SD; overweight is defined as > 1 and ≤ 2 SD and obesity is defined as > 2 SD (https://www.who.int/).

To evaluate another aging factor, we measure eotaxin-1 plasma levels in eighty-two randomly selected samples (33%) of the original subjects. Children in the STRA group [median; IQR(25-75)] [1,190 pg/mL; (108 - 2,510)] had significantly higher levels of eotaxin-1 than HC [627 pg/mL; (108 - 1,750)] (*p*<0.01) and MA [638 pg/mL; (134 - 1,460)] (*p*=0.03) (Figure 1B).

We found an inverse correlation between eotaxin-1 and rTL in the STRA group (r=-0.6, *p*=0.013) (Figure 1C). There was no correlation between rTL and other clinical and laboratory variables (data not shown).

## DISCUSSION

To the best of our knowledge, this is the first report showing a decrease in TL in children with STRA but not in MA and HC. We demonstrate that the up-regulation of the chemokine eotaxin-1 inversely correlates with TL in the STRA group but not MA or HC. Our observations are in line with previous studies in adults with chronic respiratory diseases. Cross-sectional work investigating the relationship between TL and chronic obstructive pulmonary disease (COPD) or mild and severe asthmatic adult patients [2] is related to shorter telomeres independent of cell type. In particular, Belsky et al. observed shortened TL in midlife adults with persistent childhood asthma compared with adults with adolescent or adult-onset with a comparable attrition rate, suggesting early mechanisms of DNA damage and erosion during the initial course of the disease [3]. One likely explanation for telomere erosion in STRA children could be the pro-oxidant environment. A persistent cycle of oxidative stress, DNA damage, inflammation-dependent cell-recruitment, and stem cell differentiation/repair induces tissue dysfunction and high frequency of senescent cells. ROS-dependent oxidative DNA damage has been described as an endogenous inducer for telomeres attrition [4] and a hallmark of aging. A significant source of ROS is comes from eosinophils, which respond to eotaxin-1 chemotaxis through the up-regulation of the C-C chemokine receptor type 3 (CCR3). Various cells (airway epithelial, fibroblasts, smooth muscle cells, and macrophages) secrete eotaxin-1 contributing to eosinophils recruitment. Consistent with another study in a similar cohort [5], our results show up-regulation of the eotaxin-1 gene that aggravates with worsening disease severity. Interestingly, eotaxin-1 is an age-dependent chemokine. Participants from our study had a similar age range, suggesting that STRA children exhibit a premature aging phenotype. Indeed, age-related diseases, such as asthma, display a high proportion of eosinophils, with active peroxidases propagating the pro-oxidative environment.

Ccr3 gene knockout mice have reduced production and maturation of eosinophils, homing to lung tissues, and airways hyperreactivity [6]. We found no association between rTL and frequency of eosinophils in the MA or STRA group nor with total IgE (data not shown), suggesting that at this age, eosinophil numbers might not be primarily related to telomeres shortening but subsequent actions induced by them. CCR3 is a central mediator of neuronal death. In this sense, it is plausible to speculate that the activation of the CCR3/CCL11 axis may lead to increased DNA damage, telomeres reduction, and augmented cell death in the airways. Data from our bioinformatic analyses suggest that this relationship occurs through the activation of the NADPH oxidase 1 gene (*NOX1*), which in turn leads to superoxide production (manuscript under preparation). These results are corroborated as eotaxin-1 signaling through CCR3 induces a dose-dependent increase in ROS, amplified in asthmatic patients [7]. Thus, oxidative stress might be a plausible consequence of CCL11 cellular stimulation. Furthermore, it is now known that telomeric friction occurs not only linearly during cell duplication, but also by endogenous or exogenous factors that modulate its shortening. Besides, damage caused by reactive species in telomeric regions is more difficult to recover, possibly by the interference of shelterin proteins in repair pathways [8, 9]. However, in our study, the pro-oxidant circuit was not associated with disease severity. Of note, the telomeric reduction occurs not only during cell duplication. Thus, considering the ability to increase the oxidative stress levels characteristic of the eotaxin-1 signaling and the potential of reactive species for telomeric attrition, we suggest that the association we describe in STRA occurs through a different molecular mechanism yet to be demonstrated. In STRA children, telomeres erosion might be a consequence of cumulative secretion of ROS that overwhelms the antioxidant homeostatic system leading to an accelerated aging phenotype.

## CONCLUSIONS

In summary, considering telomere length an important biological marker for the early development of many pathologies, we suggest that shorter telomeres inversely associated to augmented plasma levels of eotaxin-1 in children with STRA are indicative of a systemic senescent phenotype. Our observations support the hypothesis that severe asthma can be recognized as an aging disease present early in life.

## MATERIALS AND METHODS

### Sample characterization

One-hundred twenty-four children with MA and 17 subjects with STRA were enrolled with 126 HC children, after informed written consent from their parents and approval from the International Review Board. Asthma severity classification was done by expert physicians following American Thoracic Society (ATS) guidelines. In the MA group, 95(77%) of the subjects were receiving as-needed short-acting beta-agonist (SABA), and 29(23%) low dose of inhaled corticosteroids (ICS). In the STRA group, all patients were treated with a combination of high-dose ICS and long-acting beta-agonists (LABA), of which 7(41%) patients were also receiving omalizumab for uncontrolled persistent allergic asthma.

### Spirometry

Spirometry was done according to the ATS and European Respiratory Society (ERS) guidelines and transformed to Z-scores [10]. The spirometric parameters assessed were forced expiratory volume in 1 second (FEV1), forced vital capacity (FVC), relation FEV1/FVC and forced expiratory flow in 25 and 75% of FVC (FEF25-75%). Normalization of spirometric data, presented in Z-scores was performed through an international reference equation [11]. It was considered necessary three acceptable and two repeatable curves. After obtaining acceptable curves, reproducibility criteria were applied. Tests were repeated as far as the obtaining of repeatable values, not exceeding 8 attempts. Both the flow-volume and volume-time curves as the results were primarily analyzed by the researchers during the test; those that did not reach acceptance and reproducibility criteria were excluded at the moment of sampling [12].

### DNA extraction

Genomic DNA (gDNA) was purified from whole peripheral blood (1 mL) using UltraPure Phenol: Chloroform: Isoamyl Alcohol reagent (25:24:1, v/v, Sigma-Aldrich) and Proteinase K (2 mg/mL, Promega) as described previously [13].

### Relative telomere length

Relative mean telomere length (rTL) was performed by quantitative real-time PCR using a 96-well Real-Time PCR instrument StepOnePlus™ (Applied Biosystems). Each sample was amplified for the telomeric region (T) and a single copy (S) gene, encoding the ribosomal acid phosphoprotein P0 gene (*RPLPO*). Samples were run in triplicate (25 ng/reaction) with same spatial localization for both reactions (T or S) in different plates. T/S ratio (2ˆΔΔcT) was calculated as previously described by Cawthon with modifications [14]. Intra- and inter- variability was controlled and analyzed if cT < 0.5. Additionally, a set of 15 samples from each group was run a second time to confirm previous results and reproducibility.

### Eotaxin-1 levels

Eotaxin-1 levels were measured by enzyme-linked immunosorbent assay (ELISA) (Quantikine Human CCL11/Eotaxin Immunoassay, R&D Systems) from fresh-frozen plasma samples stored at -80° C, according to the manufacturer’s instructions.
